# Thiazolidinediones and Risk of Long-Term Dialysis in Diabetic Patients with Advanced Chronic Kidney Disease: A Nationwide Cohort Study

**DOI:** 10.1371/journal.pone.0129922

**Published:** 2015-06-17

**Authors:** Yu-Hsin Chen, Ming-Han Chiang, Jia-Sin Liu, Yu-Kang Chang, Ko-Lin Kuo, Szu-Chun Hung, Hsin-Ling Tai, Chih-Cheng Hsu, Der-Cherng Tarng

**Affiliations:** 1 Division of Nephrology, Department of Internal Medicine, Taipei City Hospital Yang-Ming Branch, Taipei, Taiwan; 2 Faculty of Medicine, National Yang-Ming University, Taipei, Taiwan; 3 Institute of Population Health Sciences, National Health Research Institutes, Zhunan, Taiwan; 4 Division of Nephrology, Taipei Tzuchi Hospital, The Buddhist Tzuchi Medical Foundation, Taipei, Taiwan; 5 Nursing Department, Taipei Veterans General Hospital, Taipei, Taiwan; 6 Department of Health Services Administration, China Medical University, Taichung, Taiwan; 7 Division of Nephrology, Department of Medicine, Taipei Veterans General Hospital, Taipei, Taiwan; 8 Department and Institute of Physiology and Institute of Clinical Medicine, National Yang-Ming University, Taipei, Taiwan; The University of Tokyo, JAPAN

## Abstract

Thiazolidinediones (TZDs) reduce urinary albumin excretion and proteinuria in diabetic nephropathy. The effect of TZDs on hard renal outcome in diabetic patients with chronic kidney disease (CKD) is unknown. We investigate the association of TZDs and risk of long-term dialysis or death in diabetic patients with advanced CKD. The nationwide population-based cohort study was conducted using Taiwan’s National Health Insurance Research Database. From January 2000 to June 2009, 12350 diabetic patients with advanced CKD (serum creatinine levels greater than 6 mg/dL but not yet receiving renal replacement therapy) were selected for the study. We used multivariable Cox regression models and a propensity score-based matching technique to estimate hazard ratios (HRs) for development of long-term dialysis and the composite outcome of long-term dialysis or death for TZD users (n=1224) as compared to nonusers (n=11126). During a median follow-up of 6 months, 8270 (67.0%) patients required long-term dialysis and 2593 (21.0%) patients died before starting long-term dialysis. Using propensity score matched analysis, we found TZD users were associated with a lower risk for long-term dialysis (HR, 0.80; 95% confidence interval [CI], 0.74-0.86) and the composite outcome of long-term dialysis or death (HR, 0.85; 95% CI, 0.80-0.91). The results were consistent across most patient subgroups. Use of TZDs among diabetic patients with advanced CKD was associated with lower risk for progression to end-stage renal disease necessitating long-term dialysis or death. Further randomized controlled studies are required to validate this association.

## Introduction

End-stage renal disease (ESRD) results in high morbidity and mortality. Dialysis has incurred significant medical and economical burdens worldwide. How to prevent impaired renal function from progressing to ESRD requiring dialysis is an important issue in the treatment of chronic kidney disease (CKD).

Diabetes mellitus is the leading cause of ESRD requiring dialysis in most countries [1,2]. In regard to optimizing glycemic control, drug choices for diabetic patients with advanced CKD are limited. It is usually not easy to convince patients to receive injection therapy. Metformin and alpha-glucosidase inhibitors are not recommended to be used in patients with estimated glomerular filtration rate (eGFR) less than 30 mL/min/1.73m^2^ [3]. Among the second line of oral antidiabetic drugs, thiazolidinediones (TZDs) are one kind of intensively used oral antidiabetics in patients with type 2 diabetes mellitus and CKD because TZDs are mainly metabolized by the liver and do not require dose adjustment in patients with renal insufficiency [3,4]. In addition to good potency on blood glucose reduction, TZDs have shown renoprotective effect in experimental models and in human studies as well [4–6]. TZDs reduce urinary albumin excretion and proteinuria in diabetic nephropathy [5,6]. However, both albuminuria and proteinuria are mere surrogates of the clinical renal end points. Till now, no study has used hard renal end points like commencing long-term dialysis to test the renal effect of TZDs.

In this study, we conducted a nationwide population-based cohort study to assess the association of TZDs and risk of long-term dialysis or death in diabetic patients with advanced CKD, serum creatinine levels greater than 6 mg/dl, but not receiving renal replacement therapy yet.

## Materials and Methods

### Data source

The study used data from the National Health Insurance (NHI) Research Database in Taiwan. The database contains health care utilization information for more than 95% of the hospitals in Taiwan and 99% of the country’s population of 23 million [7]. The information in the NHI Research Database is deidentified. We used codes from *International Classification of Diseases*, *Ninth Revision*, *Clinical Modification* (*ICD-9-CM*) to define diseases in this study (S1 Table). The study was approved by the institutional review board at Taipei Veterans General Hospital. The informed consent was waived due to the deidentified personal information in the NHI Research Database. This study complies with the Declaration of Helsinki.

### Design and study participants

**Fig 1:**
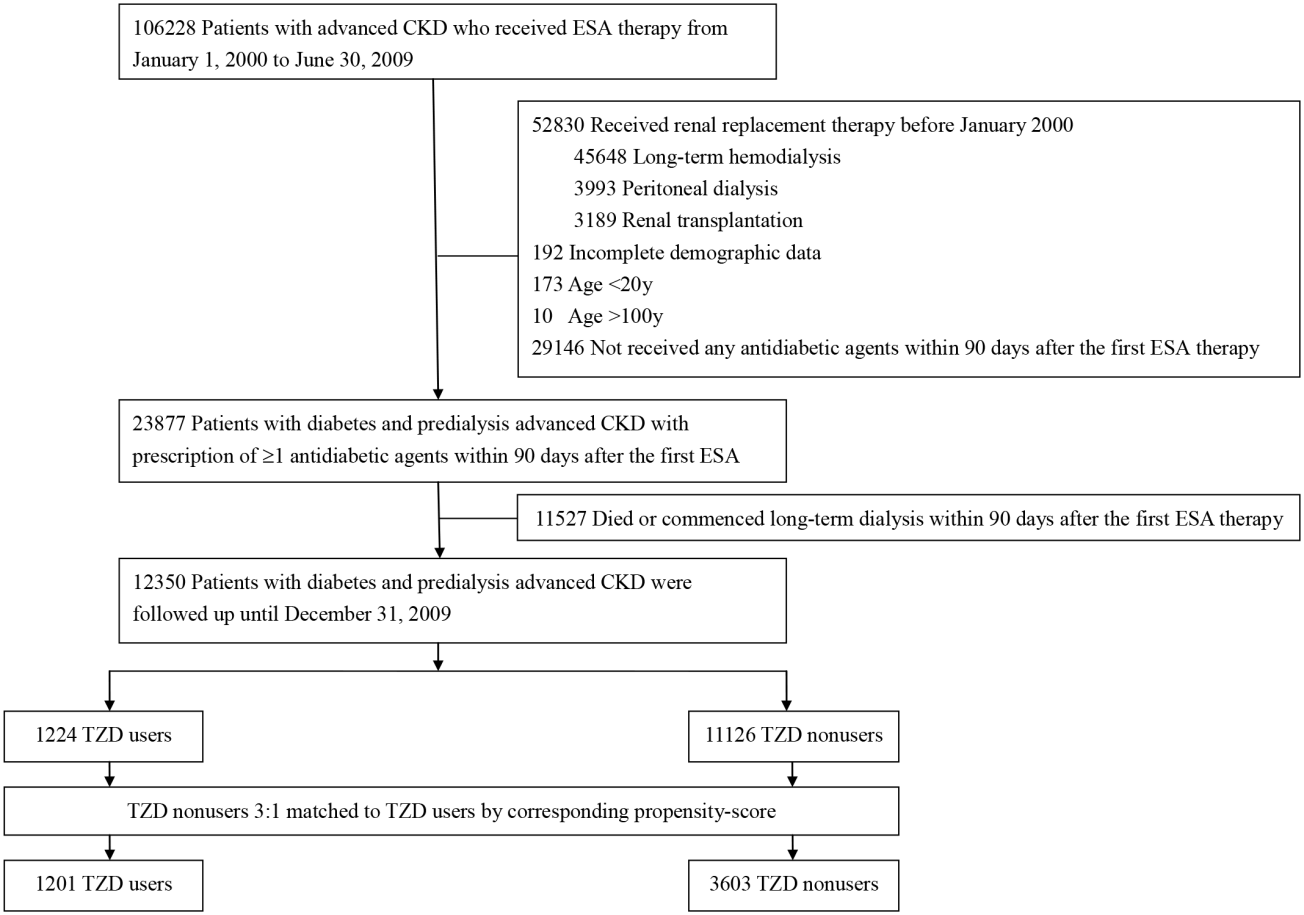
Flowchart of patient selection. CKD, chronic kidney disease; ESA, erythropoiesis-stimulating agent; TZD, thiazolidinedione.

In this nationwide population-based cohort study, we selected diabetic patients having CKD and received erythropoiesis-stimulating agent (ESA) treatment from January 1, 2000 through June 30, 2009. The present cohort represents the majority of patients with advanced CKD in Taiwan based on the NHI reimbursement regulations in Taiwan allowing patients with a serum creatinine level of greater than 6 mg/dL (approximately equivalent to eGFR less than 15 mL/min/1.73 m^2^) and a hematocrit level of less than 28% to receive treatment of ESAs. Thereafter, we excluded patients younger than 20 or older than 100 years of age and those who had received dialysis or kidney transplantation before the ESA treatment. We used prescription information within 90 days after the first ESA treatment to ascertain TZD use. The 91st day after the ESA prescription was defined as the index date. Patients who died, who commenced renal replacement therapy, or who had not been prescribed either oral antidiabetic drugs or insulin from the first ESA treatment to the index date were excluded. Patients who received kidney transplantation during follow-up period were also excluded. In Taiwan, most patients receiving kidney transplantation have already been treated with hemodialysis or peritoneal dialysis. Preemptive kidney transplant was very rare here and usually performed earlier before commencement of dialysis. Moreover, the incidence of kidney transplantation in Taiwan is very low and only 11 per million population in 2012 [8]. Accordingly, kidney transplantation is not set as a main outcome in this study although it is a form of renal replacement therapy. Finally, we selected 12350 subjects for the study (Fig 1). Comorbidities including hypertension, dyslipidemia, coronary artery disease, stroke/transient ischemic attack, heart failure, peripheral artery disease and cancer were recorded according to their *ICD-9-CM* codes (S1 Table). The Charlson comorbidity index was used to quantify patient comorbidity profiles [9]. The duration of diabetes was determined by the first time the diagnosis of diabetes (S1 Table) presented till the index date.

### Exposure assessment

Patients who had taken any TZDs within 90 days after the first ESA prescription were defined as TZD users; the remaining patients were defined as TZD nonusers. All analyses were conducted as an intention-to-treat basis according to patients’ initial TZD assignment regardless of subsequent changes to other antidiabetic regimens.

### Outcome measures

The observation period started from the index date to death, to commencement of long-term dialysis, or to December 31, 2009, whichever occurred first. The renal outcome was the commencement date of long-term dialysis. The development of the composite outcome was the start date of long-term dialysis or death, whichever came first. The events of long-term dialysis in this study were captured from the authoritative catastrophic illness registration, which was affirmed by two nephrologists.

During the observation period, we also recorded the first event of hospitalization due to diagnosis of hypoglycemia and major adverse cardiovascular events, the composite of myocardial infarction and ischemic stroke. To ascertain comparability of the intensity of diabetes control in TZD users and nonusers, we used the occurrence of hypoglycemic episodes to be a proxy index of glycemic control since the parameters of diabetes control such as blood glucose and HbA1c were not available in the NHI Research Database.

### Statistical analyses

Baseline characteristics were compared by 2-sided *t* tests and chi-square tests. Cumulative incidences for the long-term dialysis and composite outcome were generated using the Kaplan-Meier method. Between-group survival rates were compared using a log-rank test. In the multivariable Cox's proportional hazards regression models, the effects of TZDs were further adjusted for all variables listed in Table 1. A propensity score-based matching technique was used to control for residual confounding factors. For each TZD user, we identified 3 nonusers from our selected cohort who has the most similar estimated propensity score, which were calculated from all baseline covariates in Table 1. A nearest-neighbor algorithm was applied to construct matched pairs, assuming that the proportion of 0.95 to 1.0 is perfect [10]. Study entry was defined as the index date. For the renal end point of long-term dialysis, observations were censored at the end of the study or the date of death, whichever occurred first. For the composite outcome, observations were censored at the end of the study. Results were expressed as crude, adjusted, and propensity score matched hazard ratios (HRs) compared with TZD nonusers. The proportional hazard assumption, the constant HR over time, was evaluated by comparing estimated log-log survival curves for all time-independent covariates. All assessed log-log survival plots graphically showed 2 parallel lines, indicating no violation of the assumption. Propensity score matched HRs for long-term dialysis and the composite outcome of long-term dialysis or death associated with TZD use were further analyzed among subgroups, including insulin use or not, proper oral antidiabetic drugs use or not, non-steroid anti-inflammatory drugs (NSAIDs) use or not, underlying heart failure or not, and other statistically different baseline characteristics. Proper oral antidiabetic drugs excluded the patients using alpha-glucosidase inhibitor and/or metformin to minimize the unexpected effects due to inappropriate use of oral antidiabetic drugs for the patients with eGFR less than 15 mL/min/1.73 m^2^. All *P* values were 2-sided, and the significance level was set at 0.05. Analyses were performed using commercially available software (SAS, version 9.2 [SAS Institute Inc] and Stata SE, version 11.0 [Stata Corp]).

**Table 1:** Baseline characteristics of diabetic patients with advanced chronic kidney disease.

	Before Matched			Propensity Score-Matched		
	TZD	TZD		TZD	TZD	
	users	nonusers		users	nonusers	
	(n = 1,224)	(n = 11,126)	*P* value	(n = 1,201)	(n = 3,603)	*P* value
Age, mean (SD), y	65.1 (10.5)	66.1 (11.6)	0.003	65.8 (10.6)	65.7 (10.8)	0.58
Gender, female	628 (51.3)	5,599 (50.3)	0.51	586 (48.8)	1,763 (48.9)	0.93
Diabetes Duration, mean (SD), y	6.4 (2.5)	6.0 (3.2)	<0.001	6.5 (2.5)	6.5 (2.9)	0.76
Comorbidity						
Hypertension	996 (81.4)	8,974 (80.7)	0.55	976 (81.3)	2,921 (81.1)	0.88
Dyslipidemia	687 (56.1)	5,573 (50.1)	<0.001	667 (55.5)	2,005 (55.7)	0.95
Coronary artery disease	345 (28.2)	3,458 (31.1)	0.04	340 (28.3)	998 (27.7)	0.68
Stroke/transient ischemic attack	304 (24.8)	2,727 (24.5)	0.80	292 (24.3)	881 (24.5)	0.92
Heart failure	267 (21.8)	2,969 (26.7)	<0.001	267 (22.2)	793 (22)	0.87
Peripheral artery disease	34 (2.8)	287 (2.6)	0.68	31 (2.6)	99 (2.8)	0.76
Cancer	112 (9.2)	983 (8.8)	0.71	107 (8.9)	339 (9.4)	0.61
Charlson Comorbidity Index score			0.54			0.88
≤3	178 (14.5)	1,657 (14.9)		175 (14.6)	522 (14.5)	
4–5	575 (47.0)	5,041 (45.3)		567 (47.2)	1,730 (48)	
>5	471 (38.5)	4,428 (39.8)		459 (38.2)	1,351 (37.5)	
Mean(SD)	5.2 (1.9)	5.3 (2.1)	0.03	5.2 (1.9)	5.2 (1.9)	0.80
No. of nephrologist visits within 3 y before the index date			0.003			0.84
0	229 (18.7)	2,311 (20.8)		226 (18.8)	697 (19.3)	
1–6	298 (24.4)	3,299 (26.3)		295 (24.6)	858 (23.8)	
>6	697 (56.9)	5,516 (53.0)		680 (56.6)	2,048 (56.8)	
Antihypertensives used						
ACEIs	285 (23.3)	2,558 (23.0)	0.82	278 (23.2)	829 (23)	0.92
ARBs	631 (51.6)	4,638 (41.7)	<0.001	611 (50.9)	1,858 (51.6)	0.68
Alpha-blockers	351 (28.7)	3,294 (29.6)	0.50	342 (28.5)	992 (27.5)	0.53
Beta-blockers	580 (47.4)	5,475 (49.2)	0.23	569 (47.4)	1,732 (48.1)	0.68
Calcium channel blockers	919 (75.1)	8,781 (78.9)	0.002	907 (75.5)	2,685 (74.5)	0.49
Diuretics	1,030 (84.2)	8,927 (80.2)	0.001	1,009 (84.0)	3,013 (83.6)	0.75
Antidiabetic agents used						
Sulfonylureas	631 (51.6)	4,136 (37.2)	<0.001	610 (50.8)	1,859 (51.6)	0.63
Meglitinides	465 (38.0)	3,176 (28.6)	<0.001	452 (37.6)	1,347 (37.4)	0.88
Alpha-glucosidase inhibitor	255 (20.8)	1,280 (11.5)	<0.001	238 (19.8)	707 (19.6)	0.88
Metformin	161 (13.2)	844 (7.6)	<0.001	155 (12.9)	427 (11.9)	0.33
Insulin	523 (42.7)	6,523 (58.6)	<0.001	520 (43.3)	1,528 (42.4)	0.59
Statin	461 (37.7)	3,058 (27.5)	<0.001	443 (36.9)	1,299 (36.1)	0.60
Aspirin	369 (30.2)	3,223 (29.0)	0.39	359 (29.9)	1,070 (29.7)	0.90
NSAIDs						
Select	65 (5.3)	532 (4.8)	0.41	63 (5.3)	174 (4.8)	0.56
Non-select	461 (37.7)	4,448 (40.0)	0.12	455 (37.9)	1,354 (37.6)	0.85
Geographic location			<0.001			0.99
Northern	575 (47.0)	4,988 (44.8)		561 (46.7)	1,695 (47.0)	
Middle	306 (25.0)	2,337 (21.0)		297 (24.7)	893 (24.8)	
Southern	305 (24.9)	3,561 (32.0)		305 (25.4)	897 (24.9)	
Eastern or other islands	38 (3.1)	240 (2.2)		38 (3.2)	118 (3.3)	
Propensity score	0.136 (0.017–0.428)	0.095(0.010–0.500)	<0.001	0.132(0.017–0.349)	0.132(0.017–0.390)	0.79
TZDs and doses						
Pioglitazone	470 (38.4)	NA	NA			
Daily dose, mean (SD), mg	23.5 (9.9)	NA	NA			
Rosiglitazone	799 (65.3)	NA	NA			
Daily dose, mean (SD), mg	3.8 (1.9)	NA	NA			

Abbreviations: ACEIs, angiotensin converting enzyme inhibitors; ARBs, angiotensin II receptor blockers; NSAIDs, non-steroid anti-inflammatory drugs; TZD, thiazolidinedione; SD, standard deviation.

### Sensitivity analyses

We conducted additional analyses to assess the reliability of our findings. First, we conducted analyses separately for patients with index date from 2000 to 2004 and those from 2005 to 2009 to look for any evidence of a cohort effect. Second, we conducted analyses in different time windows of TZD use (i.e., within 30, 60 and 120 days after the first ESA prescription, respectively) to minimize misclassification bias. Finally, we restricted the analysis to patients receiving ESA therapy at 2 or more consecutive ambulatory care visits to exclude those under acute exacerbation of CKD with transient creatinine levels of greater than 6 mg/dL.

## Results

### Patient characteristics

We enrolled 12,350 diabetic patients with advanced CKD and anemia in this cohort study (Fig 1). Among these patients, 1224 (9.9%) had at least 1 prescription of TZD within 90 days after the first ESA prescription. Among the TZD users, 470 (38.4%) had been treated with pioglitazone and 799 (65.3%) with rosiglitazone, in which 45 patients had been used pioglitazone and rosiglitazone at different time within 90 days after the first ESA prescription. For the TZD users, their mean age was 65.1 years, 51.3% of them were women, and their mean diabetes duration was 6.4 years (Table 1). Compared with nonusers, the TZD users were younger, had longer duration of diabetes, more likely to have dyslipidemia, less likely to have coronary artery disease and heart failure. More than 40% of the selected patients were from northern Taiwan.

### Renal outcome of thiazolidinedione use

**Fig 2:**
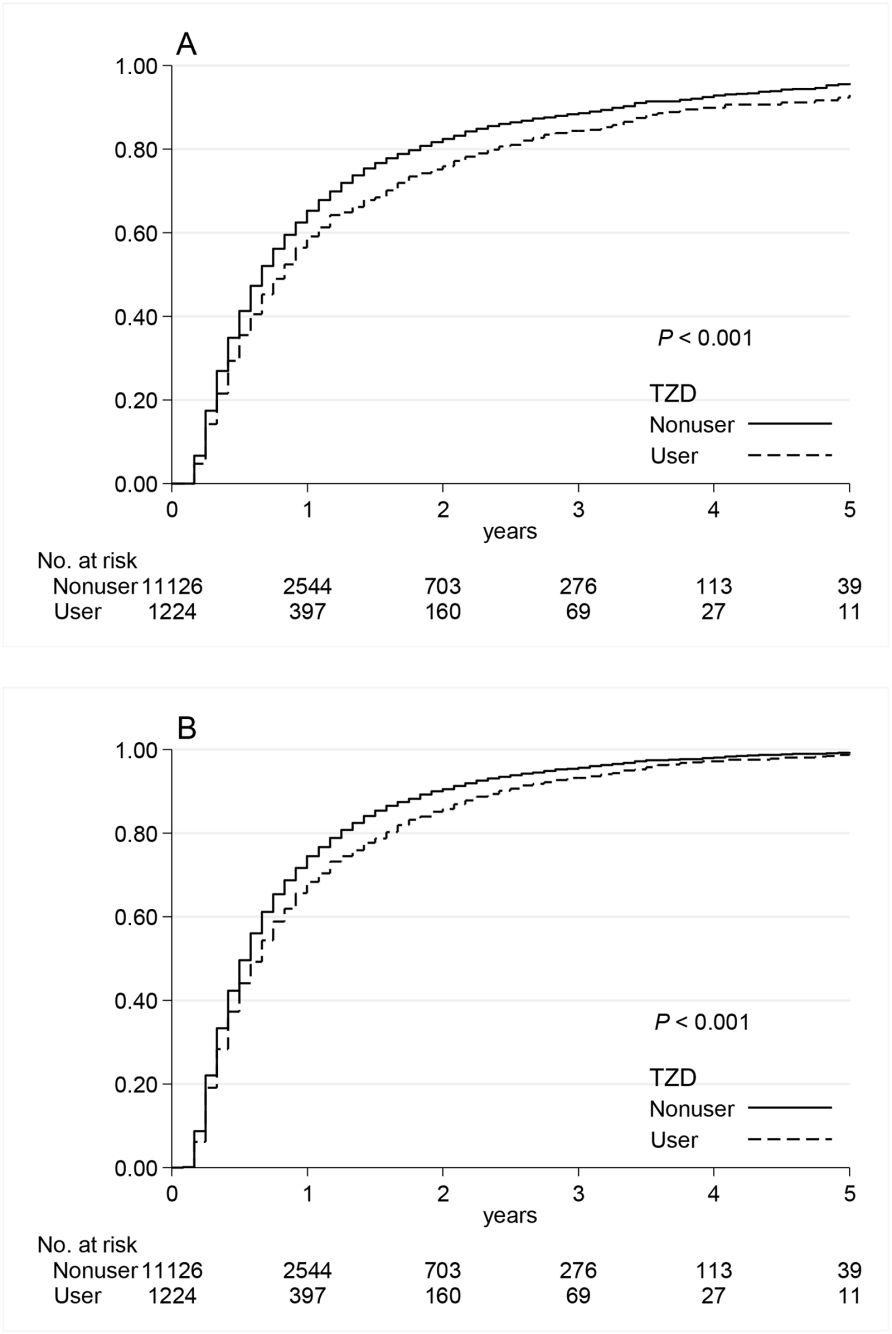
Kaplan-Meier curves of study outcomes. Cumulative incidences for long-term dialysis (a) and long-term dialysis or death (b) among diabetic patients with advanced chronic kidney disease comparing TZD users vs. nonusers. TZD, thiazolidinedione.

**Fig 3:**
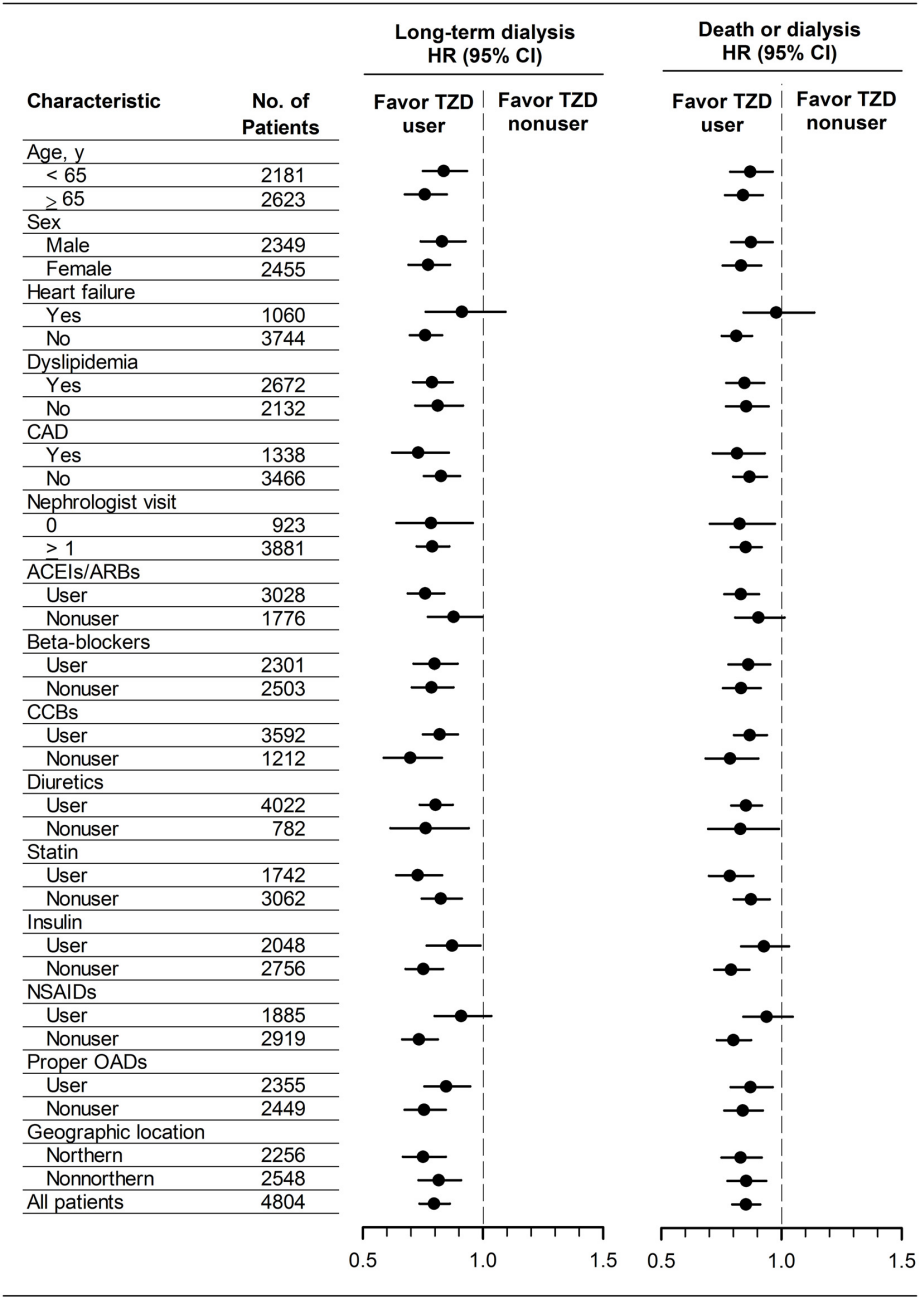
Propensity score matched hazard ratios of study outcomes among diabetic patients with advanced chronic kidney disease. Proper oral antidiabetic drugs excluded the patients using alpha-glucosidase inhibitor and/or metformin. ACEIs, angiotensin converting enzyme inhibitors; ARBs, angiotensin II receptor blockers; CAD, coronary artery disease; CCBs, calcium channel blockers; CI, confidence interval; HR, hazard ratio; NSAIDs, non-steroid anti-inflammatory drugs; OADs, oral antidiabetic drugs; TZD, thiazolidinedione.

During a median follow-up of 6 months (interquartile range, 4 to 11 months), 8270 (67.0%) patients required long-term dialysis and 2593 (21.0%) patients died before starting long-term dialysis. The relative short follow-up period resulted from the fact that patients with advanced CKD easily progress to end point of dialysis or death within 1 year (Fig 2). The number of events and incidence rates of long-term dialysis and the composite outcome of long-term dialysis or death are listed in Table 2. The Kaplan–Meier curves showed the cumulative incidences of long-term dialysis (Fig 2a) and long-term dialysis or death (Fig 2b) were both significantly lower in TZD users compared with nonusers (*P*<0.001). Table 2 demonstrated that use of TZDs in diabetic patients with advanced CKD was associated with the reduced risk for long-term dialysis with adjusted hazard ratio (HR) of 0.81 (95% confidence interval (CI), 0.75–0.87; *P*<0.001) and for the composite outcome of long-term dialysis or death with adjusted HR of 0.87 (95% CI, 0.81–0.92; *P*<0.001). The propensity score matched HRs were 0.80 (95% CI, 0.74–0.86; *P*<0.001) for long-term dialysis and 0.85 (95% CI, 0.80–0.91; *P*<0.001) for the composite outcome of long-term dialysis or death. The propensity score matched HRs of the study outcomes were also shown in subgroup analysis to minimize the residual confounding (Fig 3).

**Table 2:** Risk of study outcomes among diabetic patients with advanced chronic kidney disease comparing TZD users vs. nonusers.

	No. of Events		Incidence Rate per 100 Patient-years		Long-term Dialysis			Long-term Dialysis or Death		
Type of treatment	Long-term dialysis	Long-term dialysis or death	Long-term dialysis	Long-term dialysis or death	Crude HR (95% CI)	Adjusted HR^+^ (95% CI)	PSM HR (95% CI)	Crude HR (95% CI)	Adjusted HR^+^ (95% CI)	PSM HR (95% CI)
TZD nonuser	7,368	9656	88.4	115.8	1 (Ref.)	1 (Ref.)	1 (Ref.)	1 (Ref.)	1 (Ref.)	1 (Ref.)
(n = 11,126)										
TZD user	839	1144	70.4	96.0	0.83 (0.78–0.90)	0.81 (0.75–0.87)	0.80 (0.74–0.86)	0.85 (0.80–0.91)	0.87 (0.81–0.92)	0.85 (0.80–0.91)
(n = 1,224)										
Pioglitazone	272	377	81.0	112.3	0.91 (0.81–1.03)	0.84 (0.75–0.95)	0.84 (0.74–0.95)	0.97 (0.87–1.07)	0.96 (0.87–1.07)	0.94 (0.84–1.05)
(n = 470)										
Rosiglitzone	537	724	66.2	89.2	0.81 (0.74–0.88)	0.79 (0.72–0.86)	0.77 (0.70–0.85)	0.80 (0.75–0.87)	0.82 (0.76–0.89)	0.81 (0.75–0.88)
(n = 799)										

Abbreviations: CI, confidence interval; HR, hazard ratio; PSM, propensity score matched; TZD, thiazolidinedione.

^+^A multivariate analysis was adjusted for all variables listed in Table 1.

In sensitivity analyses, the estimated renoprotective effects of TZDs were very constant no matter if we changed the entry and observation periods (S2 and S3 Tables), redefined the exposure time for TZDs (S4–S6 Tables), or restricted analysis to patients receiving ESA therapy persistently (S7 Table). The consistent results by sensitivity analyses indicated that our findings in this study are robust.

Using hypoglycemic episodes as a proxy index of glycemic control in this study (S8 Table), we found the risk of hypoglycemia was similar in TZD user and nonuser, indicating that the intensity of glycemic control in two investigated groups might not be different.

### Hospitalization due to major adverse cardiovascular events

Table 3 demonstrates that, compared with nonusers, the TZD users were not significantly associated with increased risks for major adverse cardiovascular events (propensity score matched HR, 1.02; 95% CI, 0.83–1.26; *P* = 0.85).

**Table 3:** Risk of major adverse cardiovascular events among diabetic patients with advanced chronic kidney disease comparing TZD users vs. nonusers.

	No. of Events		Incidence Rate per 100 Patient-years		Crude HR (95% CI)	Adjusted HR^+^ (95% CI)	PSM HR (95% CI)
TZD nonuser	580	934	2.2	3.7	1 (Ref.)	1 (Ref.)	1 (Ref.)
(n = 11,126)							
TZD user	70	120	2.2	3.9	1.06 (0.87–1.28)	1.02 (0.84–1.24)	1.02 (0.83–1.26)
(n = 1,224)							

Abbreviations: CI, confidence interval; HR, hazard ratio; PSM, propensity score matched; TZD, thiazolidinedione.

^+^A multivariate analysis was adjusted for all variables listed in Table 1.

## Discussion

This nationwide cohort study first demonstrates that use of TZDs was associated with the reduced risk of hard renal outcome, i.e., long-term dialysis and composite outcome of long-term dialysis or death, among diabetic patients with advanced CKD. In this study, we showed that use of TZDs in the diabetic patients with advanced CKD might delay commencement of long-term dialysis for 3.48 months and dialysis or death for 2.14 months, which were about 30–50% improvement for the dialysis-free time (the median dialysis-free time was only 7 months for these advanced CKD patients [11]). Generally speaking, the effect of preventing long-term dialysis was consistent in the subgroup analysis. More intriguingly, the risks of hospitalization due to major adverse cardiovascular events were not significantly increased among the TZD users.

TZDs are one kind of widely used second line oral antidiabetic drugs. The major effect of TZDs, peroxisome proliferator-activated receptors (PPARs)-gamma agonists, is to improve insulin resistance by the activation of one or more PPARs. PPARs are also markedly expressed in the kidney, in the inner medullary collecting duct, interstitial cells, and in glomerular mesangial cells [12,13]. In addition to blood glucose reduction, the potential renoprotective effect of TZDs is suggested by animal studies. The possible mechanisms include blood pressure lowering, reduction of inflammatory processes, oxidative stress, lipid accumulation in mesangeal cells, level of endothelin-1, plasminogen activator inhibitor type 1 and transforming growth factor-beta; attenuation of matrix metalloproteinase-2; the stimulation of NO to improve renal endothelial function; and downregulation of renin-angiotensin system in renal vasculature [4,5].

Several human studies have examined the effect of TZDs on urine albumin excretion in patients with diabetes mellitus. Overall, in patients with normo- and microalbuminuria, treatment with TZDs significantly decreases urine albumin excretion; they can also reduce urine protein excretion in patients with frank proteinuria [6]. For the impact of TZDs on eGFR, currently available results are limited and conflicting. A retrospective study showed rosiglitazone treatment slowed the progressive deterioration of renal function in diabetic patients with an eGFR of 60–120 mL/min/1.73m^2^ and normoalbuminuria [14]. Another retrospective cohort study demonstrated rosiglitazone was associated with a decline of renal function in diabetic patients but the control group showed no renal function decline over 5 years of follow-up [15]. The *post hoc* analysis of PROactive study, which enrolled diabetic patients with documented macrovascular disease, revealed a greater decline of eGFR in pioglitazone than in control group [16]. The discrepancy between the two aforementioned studies and the present study may be because our patients were all advanced CKD and we used hard renal end point, long-term dialysis, to ascertain renal effects of TZD. The present study is the first one to investigate the hard renal outcome of TZD treatment in diabetic patients with advanced CKD.

In the cohort, sizeable proportions of patients took inappropriate drugs, such as metformin, alpha-glucosidase inhibitor, and NSAIDs. There are several reasons for the inappropriate prescription in our study subjects. First, 20.6% of the study patients never visited nephrologists within 3 years before the index date (Table 1). Second, in the clinical practice in Taiwan, patients might visit more than one doctor (a phenomenon known as doctor shopping). The inappropriate drugs for patients with CKD were possibly prescribed by doctors unaware of the patients’ CKD status. Third, in Taiwan, doctors could prescribe metformin to patients with CKD before 2009 because the Taiwan Food and Drug Administration had not limited its use in CKD until 2009. To minimize the unexpected effects due to inappropriate use of oral antidiabetic drugs and NSAIDs, we have conducted subgroup analyses by excluding those who took NSAIDs, alpha-glucosidase inhibitor and/or metformin during the study period, which might not be appropriately used in our study cohort. The results of the subgroup analyses were shown in Fig 3, indicting our findings were consistent and robust.

In subgroup analyses (Fig 3), the risk of renal outcome was reduced but not statistically significant in TZD users with heart failure, renin-angiotensin-aldosterone system (RAAS) inhibitor nonusers, and NSAIDs users. It suggested that the renal benefit of TZD treatment was possibly counteracted in these setting because RAAS blockade plays a very important protective role against the progression of advanced CKD [11] and NSAIDs are well-known for its nephrotoxicity [17].

In the present study, TZD treatment does not increase the risk of major adverse cardiovascular events in diabetic patients with advanced CKD. Pioglitazone has shown to have beneficial effect on cardiovascular diseases in patients with type 2 diabetes mellitus [18–20]. In the *post hoc* analysis from the PROactive study, diabetic patients who had eGFR less than 60 mL/min/1.73 m^2^ and documented macrovascular disease were less likely to reach a composite end point of all-cause death, myocardial infarction, and stroke when using pioglitazone [16]. In November, 2013, the U.S. Food and Drug Administration removed some prescribing and dispensing restrictions of rosiglitazone after re-evaluation of the Rosiglitazone Evaluated for Cardiovascular Outcomes and Regulation of Glycemia in Diabetes (RECORD) trial, showing no elevated risk of heart attack or death [21]. Our findings corroborated the cardiovascular safety of TZD use in diabetic patients with advanced CKD (eGFR less than 15 mL/min/1.73 m^2^).

Some limitations in this study should be acknowledged. First, the NHI Research Database provides nationwide data but some laboratory data and clinical information such as serum creatinine, eGFR, HbA1c and blood pressure are not available. Therefore, we could only combine *ICD-9-CM* codes of CKD and ESAs use to identify patients whose serum creatinine level greater than 6 mg/dL but not earlier stage of CKD. Consequently, the selected advanced CKD patients for this study had high event rate and short follow-up period. Second, confounding by treatment indication could not be totally excluded because physicians might avoid TZDs use in those who were edematous or who were likely to develop heart failure. However, the sensitivity analyses including propensity score-based matching and different cohorts (2000 to 2004 and 2005 to 2009) still showed consistently reduced risk of renal outcome among TZD users. Third, the occurrence rate of hypoglycemic episodes is merely a surrogate for glycemic control. Similarly, the rather equivalent use of anti-hypertentive medicines did not guarantee adequate blood pressure control across groups. The impact of blood pressure and blood glucose could not be totally controlled. Finally, our study is observational in nature, so it cannot prove causality.

In addition to promising efficacy of glycemic control, TZD treatment was associated with lower risks of long-term dialysis and long-term dialysis or death among diabetic patients with advanced CKD. The benefit does not come with an increased risk of major adverse cardiovascular events. Since this study is observational cohort study, further randomized controlled studies are required to validate this association.

## Supporting Information

S1 Table *International Classification of Diseases*, *Ninth Revision*, *Clinical Modification* codes used to define diseases. (DOC)

S2 Table Risk of study outcomes among diabetic patients with advanced chronic kidney disease comparing TZD users vs. nonusers, years 2000–2004. (DOC)

S3 Table Risk of study outcomes among diabetic patients with advanced chronic kidney disease comparing TZD users vs. nonusers, years 2005–2009. (DOC)

S4 Table Risk of study outcomes among diabetic patients with advanced chronic kidney disease comparing TZD users vs. nonusers, with the exposure of TZD within 30 days after the first ESA therapy. (DOC)

S5 Table Risk of study outcomes among diabetic patients with advanced chronic kidney disease comparing TZD users vs. nonusers, with the exposure of TZD within 60 days after the first ESA therapy. (DOC)

S6 Table Risk of study outcomes among diabetic patients with advanced chronic kidney disease comparing TZD users vs. nonusers, with the exposure of TZD within 120 days after the first ESA therapy. (DOC)

S7 Table Risk of study outcomes among diabetic patients with advanced chronic kidney disease comparing TZD users vs. nonusers, receiving ESA therapy persistently. (DOC)

S8 Table Risk of hypoglycemia among diabetic patients with advanced chronic kidney disease comparing TZD users vs. nonusers. (DOC)
