# Supplementary material for: Thiazolidinediones and Risk of Long-Term Dialysis in Diabetic Patients with Advanced Chronic Kidney Disease: A Nationwide Cohort Study
Source: PLoS One. 2015 Jun 17;10(6):e0129922. doi: 10.1371/journal.pone.0129922 (PMC4470911; doi:10.1371/journal.pone.0129922)
Supplement: S1 Table — (DOC) [file pone.0129922.s001.doc]

**S1 Table. *International Classification of Diseases, Ninth Revision, Clinical Modification* codes used to define diseases**

| Diseases | *ICD-9-CM* codes |
| --- | --- |
| Chronic kidney disease | 250.4*, 274.1*, 283.11, 403.*1, 404.*2, 404.*3, 440.1, 442.1, 447.3, 572.4, 580-588, 642.1*, 646.2* |
| Diabetes mellitus | 250.**, 357.2, 362.0*, 366.41 |
| Hypertension | 362.11, 401.*-405.*, 437.2 |
| Dyslipidemia | 272.* |
| Coronary artery disease | 410.**-414.** |
| Stroke/transient ischemic attack | 430-438.** |
| Heart failure | 402.01, 402.11, 402.91, 404.01, 404.03, 404.11, 404.13, 404.91, 404.93, 428.* |
| Peripheral artery disease | 443.* |
| Cancer | 140.*-208.* |
| Hypoglycemia | 251.0-251.2, 962.3 |
| Myocardial infarction | 410.** |
| Ischemic stroke | 433.**-434.** |

Abbreviations: *ICD-9-CM, International Classification of Diseases, Ninth Revision, Clinical Modification.*
