# Supplementary material for: Thiazolidinediones and Risk of Long-Term Dialysis in Diabetic Patients with Advanced Chronic Kidney Disease: A Nationwide Cohort Study
Source: PLoS One. 2015 Jun 17;10(6):e0129922. doi: 10.1371/journal.pone.0129922 (PMC4470911; doi:10.1371/journal.pone.0129922)
Supplement: S2 Table — (DOC) [file pone.0129922.s002.doc]

**S2 Table. Risk of study outcomes among diabetic patients with advanced chronic kidney disease comparing TZD users vs. nonusers, years 2000-2004^+^**

|  | Event numbers | | Incidence rate  (100 patient-years) | | Long-term dialysis | | Long-term dialysis or death | |
| --- | --- | --- | --- | --- | --- | --- | --- | --- |
| Type of treatment | Long-term dialysis | Long-term dialysis or death | Long-term dialysis | Long-term dialysis or death | Crude HR  (95% CI) | Adjusted HR  (95% CI) | Crude HR  (95% CI) | Adjusted HR  (95% CI) |
| TZD nonuser | 2729 | 3745 | 80.0 | 109.8 | 1.0 (Ref.) | 1.0 (Ref.) | 1.0 (Ref.) | 1.0 (Ref.) |
| (n = 3,747) |  |  |  |  |  |  |  |  |
| TZD user | 324 | 443 | 59.1 | 80.7 | 0.79 (0.70-0.89) | 0.74 (0.66-0.83) | 0.77 (0.70-0.85) | 0.78 (0.7-0.86) |
| (n = 446) |  |  |  |  |  |  |  |  |

Abbreviations: CI, confidence interval; HR, hazard ratio; TZD, thiazolidinedione.

^+^A multivariate analysis was adjusted for all variables listed in Table 1.
