# Supplementary material for: Thiazolidinediones and Risk of Long-Term Dialysis in Diabetic Patients with Advanced Chronic Kidney Disease: A Nationwide Cohort Study
Source: PLoS One. 2015 Jun 17;10(6):e0129922. doi: 10.1371/journal.pone.0129922 (PMC4470911; doi:10.1371/journal.pone.0129922)
Supplement: S3 Table — (DOC) [file pone.0129922.s003.doc]

**S3 Table. Risk of study outcomes among diabetic patients with advanced chronic kidney disease comparing TZD users vs. nonusers, years 2005-2009+**

|  | Event numbers | | | Incidence rate  (100 patient-years) | | | | Long-term dialysis | | | Long-term dialysis or death | | | |
| --- | --- | --- | --- | --- | --- | --- | --- | --- | --- | --- | --- | --- | --- | --- |
| Type of treatment | Long-term dialysis | Long-term dialysis or death | | Long-term dialysis | | Long-term dialysis or death | | Crude HR  (95% CI) | Adjusted HR  (95% CI) | | Crude HR  (95% CI) | | Adjusted HR  (95% CI) | |
| TZD nonuser | 4639 | 5911 | | 94.1 | | 119.9 | | 1.0 (Ref.) | 1.0 (Ref.) | | 1.0 (Ref.) | | 1.0 (Ref.) | |
| (n = 7,379) |  |  | |  | |  | |  |  | |  | |  | |
| TZD user | 515 | 701 | | 80.0 | | 108.9 | | 0.87 (0.79-0.95) | 0.85 (0.77-0.93) | | 0.91 (0.84-0.98) | | 0.92 (0.85-0.99) | |
| (n = 778) |  | |  | |  | |  |  | |  | |  | |  |

Abbreviations: CI, confidence interval; HR, hazard ratio; TZD, thiazolidinedione.

^+^A multivariate analysis was adjusted for all variables listed in Table 1.
