# Supplementary material for: Thiazolidinediones and Risk of Long-Term Dialysis in Diabetic Patients with Advanced Chronic Kidney Disease: A Nationwide Cohort Study
Source: PLoS One. 2015 Jun 17;10(6):e0129922. doi: 10.1371/journal.pone.0129922 (PMC4470911; doi:10.1371/journal.pone.0129922)
Supplement: S4 Table — (DOC) [file pone.0129922.s004.doc]

**S4 Table. Risk of study outcomes among diabetic patients with advanced chronic kidney disease comparing TZD users vs. nonusers, with the exposure of TZD within 30 days after the first ESA therapy^+^**

|  | Event numbers | | Incidence rate  (100 patient-years) | | Long-term dialysis | | Long-term dialysis or death | |
| --- | --- | --- | --- | --- | --- | --- | --- | --- |
| Type of treatment | Long-term dialysis | Long-term dialysis or death | Long-term dialysis | Long-term dialysis or death | Crude HR  (95% CI) | Adjusted HR  (95% CI) | Crude HR  (95% CI) | Adjusted HR  (95% CI) |
| TZD nonuser | 9671 | 12372 | 96.0 | 122.8 | 1.0 (Ref.) | 1.0 (Ref.) | 1.0 (Ref.) | 1.0 (Ref.) |
| (n =13,891) |  |  |  |  |  |  |  |  |
| TZD user | 912 | 1209 | 75.3 | 99.8 | 0.82(0.76-0.88) | 0.79(0.73-0.84) | 0.83(0.78-0.88) | 0.84(0.79-0.90) |
| (n = 1,278) |  |  |  |  |  |  |  |  |

Abbreviations: CI, confidence interval; HR, hazard ratio; TZD, thiazolidinedione.

^+^A multivariate analysis was adjusted for all variables listed in Table 1.
