# Supplementary material for: Thiazolidinediones and Risk of Long-Term Dialysis in Diabetic Patients with Advanced Chronic Kidney Disease: A Nationwide Cohort Study
Source: PLoS One. 2015 Jun 17;10(6):e0129922. doi: 10.1371/journal.pone.0129922 (PMC4470911; doi:10.1371/journal.pone.0129922)
Supplement: S6 Table — (DOC) [file pone.0129922.s006.doc]

**S6 Table. Risk of study outcomes among diabetic patients with advanced chronic kidney disease comparing TZD users vs. nonusers, with the exposure of TZD within 120 days after the first ESA therapy^+^**

|  | Event numbers | | Incidence rate  (100 patient-years) | | Long-term dialysis | | Long-term dialysis or death | |
| --- | --- | --- | --- | --- | --- | --- | --- | --- |
| Type of treatment | Long-term dialysis | Long-term dialysis or death | Long-term dialysis | Long-term dialysis or death | Crude HR  (95% CI) | Adjusted HR  (95% CI) | Crude HR  (95% CI) | Adjusted HR  (95% CI) |
| TZD nonuser | 6039 | 7962 | 63.4 | 83.6 | 1.0 (Ref.) | 1.0 (Ref.) | 1.0 (Ref.) | 1.0 (Ref.) |
| (n =9,311) |  |  |  |  |  |  |  |  |
| TZD user | 731 | 997 | 54.7 | 74.6 | 0.85(0.79-0.92) | 0.82(0.76-0.89) | 0.86(0.81-0.92) | 0.87(0.81-0.93) |
| (n = 1,063) |  |  |  |  |  |  |  |  |

Abbreviations: CI, confidence interval; HR, hazard ratio; TZD, thiazolidinedione.

^+^A multivariate analysis was adjusted for all variables listed in Table 1.
