# Supplementary material for: Thiazolidinediones and Risk of Long-Term Dialysis in Diabetic Patients with Advanced Chronic Kidney Disease: A Nationwide Cohort Study
Source: PLoS One. 2015 Jun 17;10(6):e0129922. doi: 10.1371/journal.pone.0129922 (PMC4470911; doi:10.1371/journal.pone.0129922)
Supplement: S7 Table — (DOC) [file pone.0129922.s007.doc]

**S7 Table. Risk of study outcomes among diabetic patients with advanced chronic kidney disease comparing TZD users vs. nonusers, receiving ESA therapy persistently^+^**

|  | Event numbers | | Incidence rate  (100 patient-years) | | Long-term dialysis | | Long-term dialysis or death | |
| --- | --- | --- | --- | --- | --- | --- | --- | --- |
| Type of treatment | Long-term dialysis | Long-term dialysis or death | Long-term dialysis | Long-term dialysis or death | Crude HR  (95% CI) | Adjusted HR  (95% CI) | Crude HR  (95% CI) | Adjusted HR  (95% CI) |
| TZD nonuser | 5,843 | 7211 | 103.3 | 127.4 | 1.0 (Ref.) | 1.0 (Ref.) | 1.0 (Ref.) | 1.0 (Ref.) |
| (n = 8,275) |  |  |  |  |  |  |  |  |
| TZD user | 674 | 844 | 86.3 | 108.1 | 0.86 (0.80-0.94) | 0.84 (0.78-0.91) | 0.87 (0.81-0.93) | 0.88 (0.82-0.95) |
| (n = 897) |  |  |  |  |  |  |  |  |

Abbreviations: CI, confidence interval; HR, hazard ratio; TZD, thiazolidinedione.

^+^A multivariate analysis was adjusted for all variables listed in Table 1.
