# Supplementary material for: Thiazolidinediones and Risk of Long-Term Dialysis in Diabetic Patients with Advanced Chronic Kidney Disease: A Nationwide Cohort Study
Source: PLoS One. 2015 Jun 17;10(6):e0129922. doi: 10.1371/journal.pone.0129922 (PMC4470911; doi:10.1371/journal.pone.0129922)
Supplement: S8 Table — (DOC) [file pone.0129922.s008.doc]

| Type of treatment | No. of Events | Incidence Rate  per 100 Patient-years | Crude HR  (95% CI) | Adjusted HR  (95% CI) |
| --- | --- | --- | --- | --- |
| TZD nonuser | 211 | 0.80 | 1 (Ref.) | 1 (Ref.) |
| (n = 11,126) |  |  |  |  |
| TZD user | 23 | 0.70 | 1.01 (0.79-1.30) | 0.86 (0.54-1.37) |
| (n = 1,224) |  |  |  |  |

**S8 Table. Risk of hypoglycemia among diabetic patients with advanced chronic kidney disease comparing TZD users vs. nonusers^+^**

Abbreviations: CI, confidence interval; HR, hazard ratio; TZD, thiazolidinedione.

^+^A multivariate analysis was adjusted for all variables listed in Table 1.
